# Circadian Clock Components Offer Targets for Crop Domestication and Improvement

**DOI:** 10.3390/genes12030374

**Published:** 2021-03-06

**Authors:** C. Robertson McClung

**Affiliations:** Department of Biological Sciences, Dartmouth College, Hanover, NH 03755, USA; c.robertson.mcclung@dartmouth.edu; Tel.: +1-603-646-3940

**Keywords:** domestication, crop improvement, circadian rhythm, circadian clock, molecular breeding, photoperiodic flowering

## Abstract

During plant domestication and improvement, farmers select for alleles present in wild species that improve performance in new selective environments associated with cultivation and use. The selected alleles become enriched and other alleles depleted in elite cultivars. One important aspect of crop improvement is expansion of the geographic area suitable for cultivation; this frequently includes growth at higher or lower latitudes, requiring the plant to adapt to novel photoperiodic environments. Many crops exhibit photoperiodic control of flowering and altered photoperiodic sensitivity is commonly required for optimal performance at novel latitudes. Alleles of a number of circadian clock genes have been selected for their effects on photoperiodic flowering in multiple crops. The circadian clock coordinates many additional aspects of plant growth, metabolism and physiology, including responses to abiotic and biotic stresses. Many of these clock-regulated processes contribute to plant performance. Examples of selection for altered clock function in tomato demonstrate that with domestication, the phasing of the clock is delayed with respect to the light–dark cycle and the period is lengthened; this modified clock is associated with increased chlorophyll content in long days. These and other data suggest the circadian clock is an attractive target during breeding for crop improvement.

## 1. Introduction

### 1.1. Plant Domestication

About 10,000 years ago, shortly after the end of the most recent ice age, humans began the transition from foraging to farming with extraordinary consequences for both the human domesticators and their domesticated plants and animals, as well as for their non-domesticated counterparts [1]. Plants were domesticated in multiple regions around the globe. For example, emmer and einkorn wheat, barley, peas, lentils, chickpeas, and flax were domesticated in the so-called Fertile Crescent of southwest Asia [1]. Rice was domesticated in China [2] and teosinte was domesticated to maize in Mesoamerica [3]. 

Plant domestication entails the exposure of wild species to new selective environments associated with human cultivation and use [1,4,5]. Although the ways in which plants are cultivated and used (for example, plants can be grown for fruits or seeds versus vegetative organs) influences the traits selected for during domestication, it is generally recognized that a common suite of traits are associated with the domestication of seed and fruit crops [1,6]. Typically, domestication is associated with increased fruit or grain size, although often the number of fruits or seeds is reduced, an increase in overall plant robustness, more determinate growth or increased apical dominance (reduced growth of side stems in comparison to the central stem), and a retention of the seeds on the plant for easy harvest. Other commonly encountered domestication traits include a loss of seed dormancy, a decrease in bitter substances in edible structures, changes in photoperiod sensitivity, and synchronized flowering [1].

Domestication is accompanied by considerable loss of genetic diversity because the farmers typically used only a limited number of individuals of the progenitor species. Moreover, only seeds from the best plants are retained for the next generation so this loss of diversity increases with each successive generation during the domestication process resulting in a genetic bottleneck. The extent of this loss of diversity depends on the population size during the domestication period and the duration of that period [7]. However, genetic diversity is not lost equally throughout the genome [1]. For genes that influence the desirable phenotypes (like those described above) the loss of diversity is greater because plants carrying selected alleles contribute more progeny to each subsequent generation and other alleles are reduced in frequency or eliminated from the population. In contrast, the loss in diversity of genes that do not influence favored phenotypes (neutral genes) is less and simply a function of the strength of the bottleneck in terms of the population size and duration [1,8].

Here, I adopt the terminology of Meyer and Purugganan [9] and define a domestication gene as a gene for which function has been characterized and underlies a trait that has undergone positive selection, and for which the causative mutation is completely or near-completely fixed in all lineages from that single domestication event [9]. Other genes that control important traits but for which causative mutation(s) are segregating in domesticated populations are considered as diversification or improvement genes that played a lineage-specific role in the crop’s regional adaptation or subsequent improvement. Initial domestication occurs in the environment of the wild progenitor. However, following this initial domestication, there is frequently a period of range expansion in which successfully domesticated crops are moved through trade into new geographic regions where they encounter novel environmental features, both abiotic and biotic. This range expansion, therefore, can be expected to be accompanied by selection for improved performance in these novel environments. The circadian clock plays an important role in the response of plants to their environment and therefore is a likely target for selection during this second period of diversification or improvement [10]. The purpose of this review is to consider the evidence that such selection for altered clock function has occurred.

### 1.2. The Plant Circadian Clock

#### 1.2.1. The Plant Circadian Clock Consists of Multiple Interlocked Feedback Loops

The rotation of the earth on its axis means that life in the biosphere is exposed to a daily cycle of light and relative warmth versus dark and relative cold. This drastic daily change in the environment occurs with a predictable 24-h period and a fitness advantage is conferred by the ability to anticipate those environmental changes and adjust physiological and metabolic states in coordination with the expected environmental conditions [11,12,13]. Thus, circadian clocks can be found in all domains of life, including Archaea, Bacteria, and Eukaryota [14]. Within the Eukaryota, circadian clocks found in animals, fungi, and plants all have a common architecture of interlocked feedback loops, although the molecular components that comprise plant clocks do not seem to be evolutionarily related to those in animal and fungal clocks [15]. However, plant clocks seem to be complex and include more feedback loops than animal and fungal clocks. This may be a consequence of a proliferation of clock genes resulting from the multiple instances of polyploidization that have occurred during plant evolutionary history [16].

In plants, the oscillator mechanism central to circadian rhythm generation consists of multiple interlocked transcription/translation feedback loops [17,18]. Proteins within the oscillator provide feedback by directly or indirectly repressing their own activity or their own transcription. One cycle of activation and repression of these proteins, followed by activation again, takes ~24 h and generates the period of the circadian rhythm. Although transcriptional activation and repression contribute critically to plant clock function, many post-transcriptional regulatory mechanisms also play integral roles. For example, alternative splicing has been shown to affect circadian timing [19,20,21] and mutations affecting spliceosomal components and their modification alter circadian period [22,23,24,25]. Protein synthesis, protein post-translational modification, protein stability, and protein subcellular localization also play important roles in clock function [26,27]. For example, after dusk ZEITLUPE (ZTL), an F-box protein with a blue light photosensing LOV (Light, Oxygen, Voltage-sensitive) domain, targets PRR5 and TOC1 for ubiquitylation and proteasomal degradation [28,29,30,31,32,33,34,35].

Most of our knowledge of the plant circadian clock has been gained through studies in Arabidopsis. Many of the components of the Arabidopsis circadian oscillator have been identified, although our understanding of the circadian oscillator remains incomplete and new components and regulatory relationships continue to be identified and characterized; e.g., [36,37]. The first identified plant circadian clock component was TIMING OF CAB EXPRESSION1 (TOC1), so-named because mutations that impair TOC1 function shorten the period of the circadian rhythm as assayed using the *CAB2:LUC* reporter [38]. The abundances of both *TOC1* mRNA and TOC1 protein show circadian rhythms, with mRNA abundance maximal at about dusk and protein abundance maximal at night [39]. When *TOC1* is expressed constantly using a heterologous promoter, all circadian rhythms tested are abolished, demonstrating that rhythmic expression of TOC1 is essential for function of the circadian clock [40]. *TOC1* encodes a nuclear protein with sequence motifs similar to those found in two-component signal-transduction systems common in bacteria and functions as a transcriptional repressor [39,41].

Two other oscillator proteins are the nuclear MYB-related transcription factors LATE ELONGATED HYPOCOTYL (LHY) and CIRCADIAN CLOCK ASSOCIATED 1 (CCA1). These are closely related proteins whose abundance shows circadian rhythms, peaking at dawn. Mutations that impair the function of CCA1 or LHY cause circadian rhythms to cycle with a short period, and inactivating both genes dramatically shortens period, indicating that these two proteins function at least partially redundantly. As with *TOC1*, constant expression of either *CCA1* or *LHY* causes arrhythmicity, establishing the necessity of cycling expression of CCA1 and LHY for circadian clock function [42,43,44,45].

A model was proposed in which LHY and CCA1 interact with TOC1 to create a negative autoregulatory feedback loop at the heart of the plant circadian oscillator [46]. LHY and CCA1 begin to accumulate just before dawn and repress *TOC1* expression and eventually, as their proteins accumulate, repress their own expression. As the levels of LHY and CCA1 proteins fall, the expression of *TOC1* mRNA rises, with a peak of expression at the end of the day. TOC1 protein then indirectly activates the expression of the *LHY* and *CCA1* genes, thereby starting another cycle.

**Figure 1:**
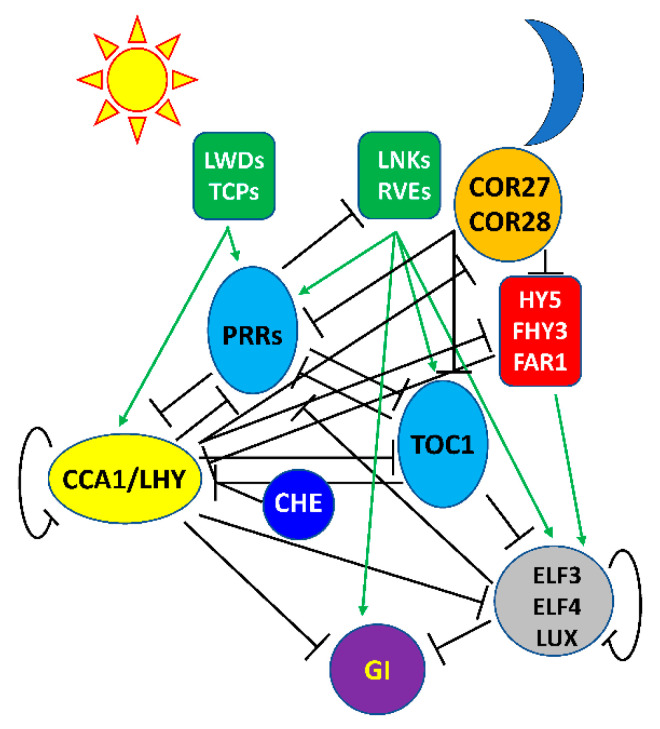
A simplified representation of the Arabidopsis circadian clock network. The clockwork components are represented from left to right according to the time-of-day of their peak expression. CCA1 and LHY are expressed early in the morning, followed sequentially by PRR9, PRR7, PRR5, and TOC1, which peaks at dusk. The “evening complex” (EC) is formed by LUX, ELF3, and ELF4, and is expressed after dusk. These components are transcriptional repressors, with repression indicated by lines ending in perpendicular lines. TOC1 interacts with CHE (TCP21) to negatively regulate *CCA1* and *LHY*. In the morning, the transcription factors TCP20 and TCP22 with LWD1 and LWD2 acting as coactivators activate expression of (indicated by the green arrows) *CCA1*, *LHY*, *PRR9* and *PRR5*. In the middle of the day RVE8 (and RVE4 and RVE6), with LNK1 and LNK2 acting as coactivators, activate expression of *PRR5*, *TOC1*, *GI*, *LUX*, and *ELF4*. Late in the day. FHY3, FAR1, and HY5 activate *ELF4* expression. Please consult the main text for further details.

It soon became clear that this simple model was incomplete; we now know that the Arabidopsis circadian clock is much more complex, with more than 20 transcription factors assembled into multiple interlocked feedback loops (Figure 1). CCA1/LHY are the first in a progression of transcriptional repressors expressed sequentially over the day: CCA1/LHY expression is followed by that of a family of PSEUDO RESPONSE REGULATORS (PRRs) closely related to TOC1 in the sequence of PRR9, PRR7, and PRR5 [18,47,48,49] and then TOC1 at dusk. TOC1 is a transcriptional repressor and interacts with TEOSINTE BRANCHED1-CYCLOIDEA-PCF21 (TCP21, also called CHE) to repress CCA1 [50]. The other PRRs are also transcriptional repressors, interacting with the transcriptional corepressor TOPLESS (TPL) through a conserved EAR motif [51]. Recently it has also been shown that COLD-REGULATED 27 (COR27) and COR28 repress *PRR5* and *TOC1* expression [36,37]. COR27 and COR28 lack DNA-binding activity and likely serve as co-repressors by interacting with as-yet unidentified DNA-binding transcription factors. *COR27* and *COR28* expression is repressed by CCA1 [37]. The Evening Complex (EC), consisting of LUX ARRHYTHMO (LUX) or the close LUX homolog, BROTHER OF LUX ARRHYTHMO (BOA, also known as NOX), complexed with EARLY FLOWERING 3 (ELF3) and ELF4, accumulates after dusk [52,53,54]. The EC and all of these other components function as transcriptional repressors and each represses expression of the previous and subsequent components in the progression. The EC accumulates after dusk and maintains repression of *CCA1* and *LHY*, restricting their expression to late night and early morning.

Much less is known about transcriptional activators in the circadian oscillator, likely due to functional redundancy hindering their identification via loss of function mutations. Expression of *CCA1* and *LHY* is activated around dawn by a complex of the LIGHT-REGULATED WD1 (LWD1) and LWD2 with DNA-binding TCP20/TCP22 transcription factors [55]. Consistent with a role in the regulation of CCA1 transcription, TCP20 transcript cycles with a pre-dawn maximum [56]. The LWD/TCP complex also activates expression of the *PRRs*. Later in the afternoon a complex (or complexes) of REVEILLE (RVE) transcription factors (RVE8, RVE4, and RVE6) closely related to CCA1 and LHY together with members of the family of NIGHT LIGHT–INDUCIBLE AND CLOCK-REGULATED (LNK) transcriptional corepressors activate the expression of *PRR5*, *TOC1*, *GIGANTEA* (*GI*), and *ELF4* [57,58,59,60], although expression of the EC remains repressed until after the degradation of PRR5 and TOC1. *ELF4* transcription is also activated by FAR-RED ELONGATED HYPOCOTYL3 (FHY3), FAR-RED IMPAIRED RESPONSE1 (FAR1), and ELONGATED HYPOCOTYL5 (HY5), three transcription factors that are positive regulators of phytochrome A signaling [61]. FHY3 and FAR1 also directly bind to the *CCA1* promoter to drive light-induced *CCA1* expression [62]. Interestingly, CCA1 and LHY interact with and inhibit the transcriptional activation activity of FHY3, FAR1, and HY5, which contributes to the roles of CCA1 and LHY as transcriptional repressors [61]. Similarly, COR27 interacts with HY5 to repress its DNA binding activity [63].

It is not surprising that these rhythmic changes in transcription of clock genes are accompanied by changes in chromatin structure. This was first described for *TOC1*, where histone acetylation/deacetylation cycles with transcriptional activity [64]. The EC interacts with HISTONE DEACETYLASE9 (HDAC9) to recruit it to the TOC1 promoter to repress TOC1 expression at night [65]. The EC and HDA9 also interact with HIGH EXPRESSION OF OSMOTICALLY RESPONSIVE GENE15 (HOS15) to deacetylate histones at the *GI* promoter and transcriptionally repress *GI* expression at night [66]. There are multiple additional examples of epigenetic control of clock gene expression that have been recently reviewed as part of this Special Issue [67].

#### 1.2.2. Photoperiodic Induction of Flowering

Successful reproduction is essential for fitness, so the timing of flowering to ensure maximal reproductive success is subject to natural selection in wild species and likewise has been subject to artificial selection in domesticated species. Flowering can be initiated in response to both environmental cues and endogenous pathways, although their relative importance varies among species. Here, I focus on photoperiodic flowering. Photoperiodism makes it possible for plants to infer seasonality from day length. At the equator, day length and night length are equal and remain constant throughout the year. However, as one moves from the equator towards the poles, the days become longer in summer and shorter in winter. Plants detect these seasonal changes in day length and use them to coordinate their flowering to the appropriate season.

**Figure 2:**
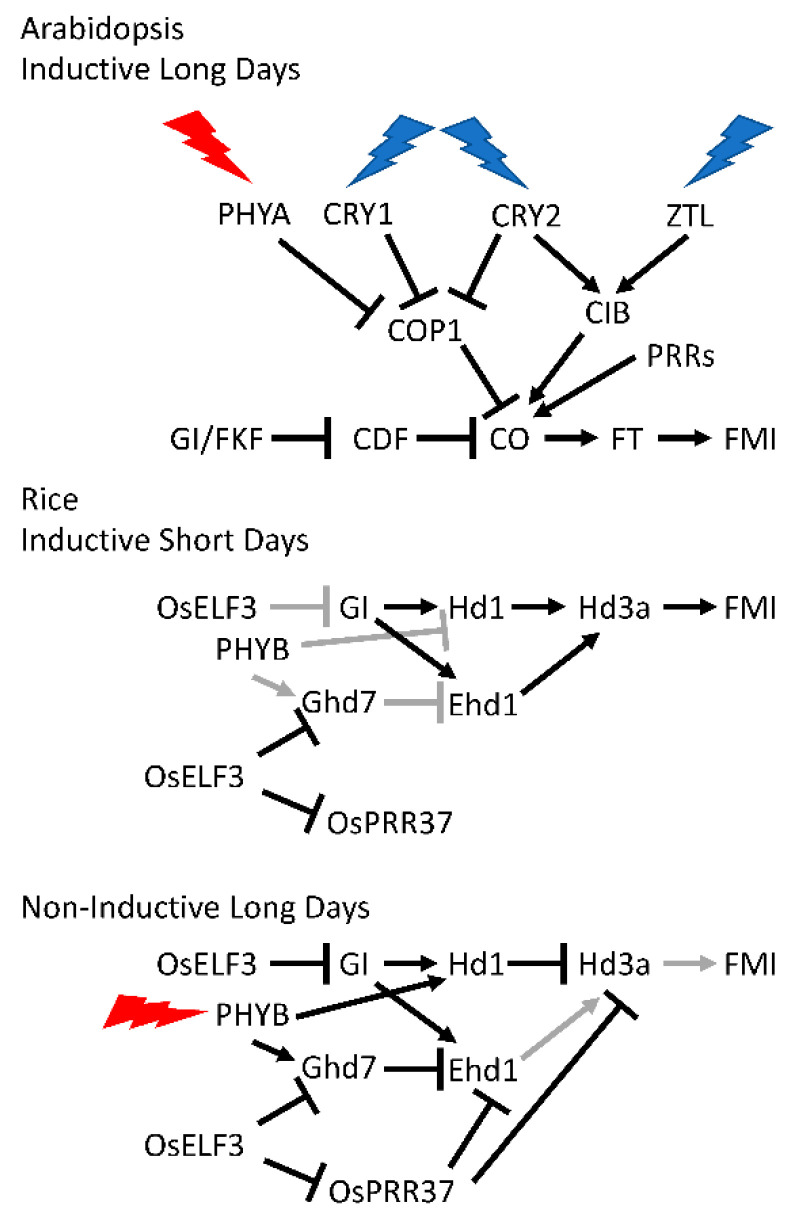
Regulation of photoperiodic flowering in Arabidopsis, a long-day plant, and in rice, a short-day plant. Upper panel. In Arabidopsis, CO is a central activator of flowering time, inducing expression of *FT*. FT protein acts as florigen, traveling from the leaf vasculature to the shoot apical meristem where it, together with FD, induces expression of the *FLORAL MERISTEM IDENTITY* (*FMI*) genes. *CO* transcription is repressed by the CDF proteins, which in long days are targeted for proteolytic degradation by the LOV/KELCH/F-BOX protein FKF1 complexed with GI. *CO* transcription is also independently activated by light-activated CIB proteins. In long days light signaling stabilizes CO protein through inhibition of proteolytic degradation of CO, which in short days is ubiquitylated by the E3 ubiquitin ligase COP1. CO protein is also stabilized through interaction with the PRRs during the day, when they are maximally abundant. Please consult the main text for further details. Middle panel. In rice in inductive short days the OsGI/Hd1 (OsCO) pathway activates expression of Hd3a (OsFT). Hd3a expression is also activated by Ehd1 in a distinct pathway not found in Arabidopsis. Black indicates strong regulation and gray weak regulation. Please consult the main text for further details. Lower panel. In rice in non-inductive long days light signaling through PHYB converts Hd1 into a transcriptional repressor of Hd3a. Light signaling also induces Ghd7 which encodes a repressor of Ehd1 expression. Similarly, OsPRR37 represses Ehd1 and Hda3 expression. Black indicates strong regulation and gray weak regulation. Please consult the main text for further details.

Typically, the leaf is the site of perception of the photoperiodic signal, generating a mobile flowering inducer, termed florigen, that is transmitted to the shoot apical meristem where it induces *FLORAL MERISTEM IDENTITY* (*FMI*) genes, the key regulators that initiate the genetic programs required for flower development [68]. This pathway has been worked out in considerable detail in Arabidopsis, in which flowering is accelerated in response to long days (Figure 2). In Arabidopsis, florigen is encoded by the *FLOWERING LOCUS T* (*FT*) gene. The circadian clock regulates the induction of *FT* and of its critical transcriptional inducer, CONSTANS (CO), via an external coincidence mechanism in which light coincides with an inductive window that is restricted (gated) by the circadian clock [69,70].

The circadian clock drives morning-specific expression of several *CYCLING DOF FACTOR* (*CDF*) genes whose protein products repress *CO* transcription. The CDF proteins are targeted for degradation by a SCF complex containing FLAVIN BINDING, KELCH REPEAT, F-BOX1 (FKF1) and the clock component GIGANTEA (GI), both of which cycle in protein abundance. In short days, GI protein abundance peaks at dusk while FKF1 protein peaks after dark. The FKF1-GI complex forms in the dark and only degrades the *CO*-repressing CDF proteins after dusk. Thus, *CO* transcription is repressed until about dusk and *CO* mRNA accumulates after dusk. CO protein is unstable in the dark so, in short days, CO protein fails to accumulate and *FT* transcription is not induced. However, in long days the phase of peak GI accumulation coincides with that of FKF1 before dusk. The FKF1-GI complex degrades the CDFs in the late afternoon, relieving transcriptional repression of *CO. CO* mRNA accumulates in the light, which permits the stabilization of nascent CO protein and activation of *FT* transcription. In this way *FT* is expressed in the vasculature of the leaf under inductive photoperiods and FT protein travels through the phloem to the shoot apical meristem where it works together with meristem expressed FD to induce *FLORAL MERISTEM IDENTITY* (*FMI*) genes and initiate flowering.

A critical element of this photoperiod pathway is the light-mediated stabilization of CO protein. CO protein is degraded via COP1 in the dark, but in the light, PHYA, CRY1, and CRY2 suppress COP1 activity to stabilize CO [70,71,72]. Similarly, the PRRs physically interact with and stabilize CO protein during the day, when they are abundant [73].

*FT* transcription is also induced independently of CO. Several CRY2-INTERACTING bHLH (CIB) transcription factors accumulate in long days to stimulate *FT* transcription. The CIBs are activated in the afternoon by blue-light-dependent interaction with CRY2. In addition, CIB protein stability is enhanced via a blue-light-dependent interaction with the FKF1 relatives, ZTL and LKP2, although not with FKF1 [70,74,75].

In crops, flowering time is an important agronomic trait that determines seasonal and regional adaptation. If a cultivar flowers too early in a specific location there will be inadequate use of light and temperature resources, and consequent lower yield. On the contrary, if a cultivar is too late in flowering it cannot complete flowering and grain development before the onset of cold, also resulting in lower yield.

Let us consider flowering time (heading date) in rice, a cereal, as an example (Figure 2). The CO-FT pathway is important in photoperiodic flowering in rice, a short-day plant, but additional pathways also play important roles [70,76]. As in Arabidopsis, florigens encoded by the rice *FT* homologs, *Heading Date 3a* (*Hd3a*) and *RICE FLOWERING LOCUS T 1* (*RFT1*), are induced in the leaf vasculature under inductive (short) days and move to the shoot apical meristem to induce flowering. Hd1, the rice CO ortholog, promotes *Hd3a* transcription under short days. However, in long days Hd1 is converted from an activator to a repressor by light signaling from phytochrome B and inhibits *Hd3a* transcription [77,78]. There is also a second bifunctional transcriptional regulator of *Hd3a* that activates in short days and represses in long days. Thus, although there are general similarities with the Arabidopsis CO-FT pathway, there are clear differences in mechanistic detail [70].

Rice has a second pathway that regulates *Hd3a* expression. Early heading date 1 (Ehd1), a rice-specific B-type response regulator, upregulates *Hd3a* expression to promote flowering mainly in short days [79], when blue light signaling coincides with the morning phase set by the circadian clock. *Ehd1* expression is inhibited by Ghd7 (a CCT-domain protein encoded by *GRAIN NUMBER, PLANT HEIGHT AND HEADING DATE 7*), which is regulated by the circadian clock [80,81,82]. *OsELF3-1*/*Hd17*/*Early flowering7* (*Ef7*) participates in this repressive regulation of *Ghd7* [83,84,85]. Disruption of *OsELF3-1*/*Hd17*/*Early flowering7* (*Ef7*) function results in elevated expression of *Ghd7* in both long and short days, resulting in reduced *Ehd1* and *Hd3a* expression [84,85]. *OsELF3-1*/*Hd17*/*Early flowering7* (*Ef7*) also negatively affects *OsGI* expression, which is responsible for *Ehd1* expression, and *OsPRR37*, which suppresses expression of *Hd3a* but not that of *RFT1* under long-day conditions [83,84,85]. OsPRR37 functions as a transcriptional repressor expression of day-phased clock genes as well as *Ehd1* [86].

Photoperiodic flowering in the long-day cereals, wheat and barley, has been reviewed elsewhere [70] and will not be addressed here due to space limitations.

## 2. Circadian Clock Components as Domestication and Crop Improvement Loci

As mentioned above, plants were domesticated in a number of distinct geographical regions [1] and it is self-evident that initial domestication occurred in the environment of the wild progenitor. However, many domesticated crops were subsequently moved into new geographic regions where they encountered novel environmental conditions.

Environmental changes associated with range expansion include those in the annual patterns of temperature and photoperiod, both of which vary with latitude [87]. As a consequence, in a number of animals circadian clock function varies systematically with latitude. For example, in *Drosophila melanogaster* alternative splicing of the clock gene *period* (*per*) alters an activity rhythm under warmer temperatures to avoid desiccation [88]. Latitudinal clines are found in two predominant splice forms of *per* that alter temperature compensation of the clock [89,90,91]. Similarly, latitudinal clines in the frequencies of allelic variants of the circadian rhythm gene *Clock* are found in birds (*Cyanistes caeruleus*) [92] and salmon (*Oncorhynchus tshawytscha*) [93]. This establishes that divergent selection on circadian clock function contributes to local adaptation in animals.

Are similar latitudinal clines in clock function found in plants? Initial studies in Arabidopsis found suggestive correlations of period with latitude [94,95]. A positive correlation was observed between latitude and circadian period in the wildflower, *Mimulus guttatus* [96]. One might therefore expect to find alterations in clock function that accompany latitudinal expansion in crops.

### 2.1. Soybean

One phenotype likely to respond to latitude is photoperiodic flowering, and soybean offers a number of excellent examples. Soybean (*Glycine max*) was domesticated from wild soybean (*Glycine soja*) in East Asia 6000–9000 years ago [97,98]. Soybean is a short-day plant that flowers when daylength becomes shorter than a critical length [99]. Photoperiod-sensitivity determines the cultivation limits of soybean, making control of flowering time important for regional adaptation and range expansion [100,101,102]. Multiple maturity loci, including 11 *E* loci, have been identified [103,104,105]. Dominant alleles at *E1*, *E2*, *E3*, *E4*, *E7*, *E8*, and *E10* confer late flowering, whereas dominant alleles at *E6*, *E9*, and *E11* confer early flowering [102]. Of these *E* loci, E2 is *GmGI* [106], *E3* is *GmPHYA3* [107], and *E4* is *GmPHYA2* [108], all genes with roles in clock oscillator function or in light input to the clock in Arabidopsis. Allelic variation among these *E* loci is associated with differences in flowering time and adaptation to different latitudes among soybean cultivars [102,109,110,111,112,113,114,115,116,117].

There are a total of 12 *FT* homologs in soybean [102]. *E9* is *GmFT2a* [118]. *GmFT2a* and *GmFT5a*, which are highly induced under inductive short-day conditions, are the most important flowering inducers [99]. These genes are not fully redundant and play distinct roles in floral induction, with loss-of-function of *GmFT2a* associated with delayed flowering under SD and loss-of-function of *GmFT5a* associated with delayed flowering under LD [119,120]. *GmPHYA3* and *GmPHYA2 (E3* and *E4*, respectively), are negative regulators of both *GmFT2a* and *GmFT5a* under non-inductive long-day conditions [99]. *GmFT1a*, *GMFT1b*, *GmFT4*, and *GmFT6* act as floral inhibitors, like *TFL1* in Arabidopsis [121,122,123,124]. *E10* most likely corresponds to *GmFT4* [104]. It remains to be determined the extent to which either standing or induced variation among these many *FT* homologs can contribute usefully to the manipulation of photoperiodic flowering towards improved field performance.

During post-domestication improvement, soybean, a short-day plant, has gained the ability to flower under long-day conditions during the growing season at higher latitudes. This has come about mainly through dysfunction of *GmPHYA3* and *GmPHYA2* and the floral repressor *E1* [103]. Recently, photoperiod insensitivity and earlier flowering in long-day conditions in Far-Eastern Russian soybean cultivars was associated with loss of function of *E1-Like b* (*E1Lb*), a homeolog of *E1* [125]. Lines carrying the loss-of-function *e1lb* allele exhibited elevated expression of *GmFT2a* and *GmFT5a* and flowered earlier than those carrying the functional *E1Lb* allele under long-day conditions. Thus, *E1Lb* retards flowering under long-day conditions by repressing the expression of *GmFT2a* and *GmFT5a* independently of *E1* [125]. A second *E1-Like* gene, *E1La*, also functions as a floral repressor [126]. Early maturity is critical for northern expansion of soybean cultivation in the USA and Canada as well as in northeast Asia. *GmFT5a* has also been shown to underlie a QTL promoting flowering under long days [127].

*GIGANTEA* (*GI*) was first identified in Arabidopsis through loss of function mutations that were very late flowering, allowing prolonged vegetative growth, which explains the name of the locus [128]. In long-day plants such as *Pisum sativum*, *Hordeum vulgare*, *Triticum aestivum*, and *Brassica rapa*, as in Arabidopsis, *GI* acts as a flowering activator [129,130,131,132]. In contrast, in soybean and rice, both short day plants, *GI* acts as a floral repressor [81,106,133]. In soybean, there are three *GI* loci, *GmGIa*, *GmGIb* (*GmGI1*), and *GmGIc* (*GmGI2*), although only *GmGIa*, which corresponds to *E2*, has been established to be important in flowering and maturity [106]. However, both *GmGIb* (*GmGI1*), and *GmGIc* (*GmGI2*) bind to both GmFKF1 and GmFKF2 and to GmCDF1 and so may contribute to flowering time regulation [134]. Nonetheless, there has been no loss of nucleotide diversity of *GmGIb* and *GmGIc* in domesticated relative to wild soybean, indicating that they have not been subject to selection during domestication and improvement [135]. In contrast, there has been a great loss in diversity of *GmGIa* during domestication and improvement [135]. In particular, among Chinese genotypes there has been enrichment for a haplotype that encodes a truncated GI protein and presumably confers at least partial loss of function, permitting earlier flowering in long days. Interestingly, this haplotype was not found among Japanese and Korean wild soybeans [135], although another haplotype with a nonsense mutation in the second exon has been found in Korean early flowering lines [136].

In Arabidopsis it is well-established that GI regulates photoperiodic flowering through an external coincidence mechanism in which in long days the phase of peak *GI* expression coincides with that of *FKF1* in late afternoon [70]. FKF1 is a blue-light photoreceptor, and the interaction of FKF1 with GI is enhanced by blue light [137]. The resultant FKF1-GI complex degrades the CDFs, transcriptional repressors of *CO*, in the late afternoon of long days [138]. Thus, *CO* mRNA accumulates in the light, which permits the stabilization of nascent CO protein and consequent activation of *FT* transcription [139]. However, GI also exerts clock-independent effects on flowering. In particular, Arabidopsis GI positively regulates transcription of *miR172* [140], which promotes photoperiodic flowering through a CO-independent genetic pathway. *miR172* post-transcriptionally represses a set of *APETALA2* (*AP2*)-like genes, including *TARGET OF EAT1* (*TOE1*), *TOE2*, and *TOE3* that repress *FT* transcription [140,141]. Overexpressing TOE1 causes late flowering, whereas *miR172*-overexpressing plants exhibit early flowering under both long and short days [140]. As in Arabidopsis, soybean GmGIa positively regulates *Gma-miR172a,* although through post-transcriptional processing [142]. *Gma-miR172a* targets include a soybean *TOE1* ortholog, Glyma03g33470. Thus, GmGIa serves as a photoperiod-independent flowering activator by increasing the expression of TOE1 targets, including *FT*, *AP1* and *LFY* [142].

Photoperiodic responses have also hampered the expansion of soybean cultivation into the tropics because temperate varieties matured extremely early, leading to a reduced vegetative size that resulted in extremely low grain yields when temperate lines were grown below 20° latitude. Long juvenile phase (LJ) soybean lines discovered in the 1970s exhibit delayed flowering under short photoperiods, which significantly enhanced yield [143,144]. Two loci, *J* and *E6*, have been reported to control this response [145,146]. *E6* has been mapped and is tightly linked to *J* [147]. Genetically, *E6* acts as a suppressor of *E1* and the function of *E6* is dependent on *E1*. However, the molecular identify of *E6* remains to be established [147].

**Figure 3:**
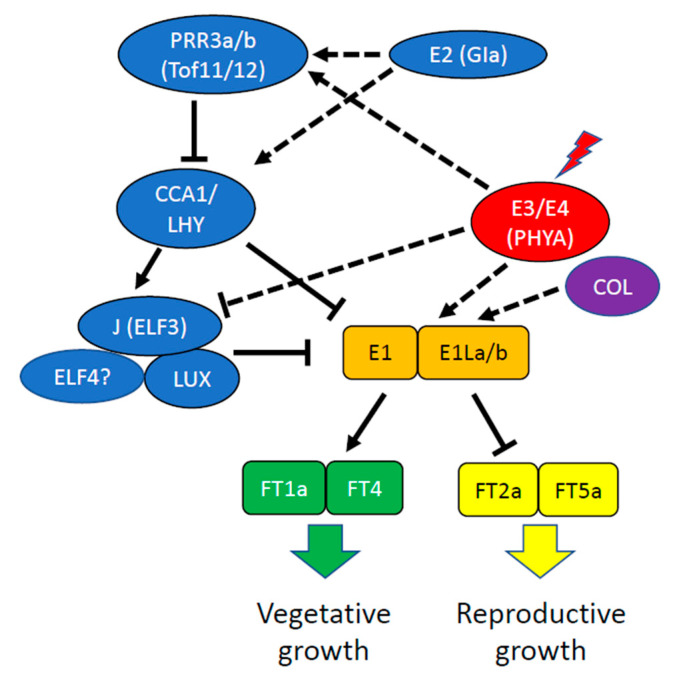
Regulation of photoperiodic flowering in soybean. In inhibitory long days, red light signaling through PhyA, encoded by *E3* and *E4,* induces the expression of the flowering repressors, E1, E1La and E1Lb. E1, E1La and E1Lb inhibit expression of *FT2a* and *FT5a*, which encode florigens that travel to the shoot apical meristem to induce *FLORAL MERISTEM IDENTITY* genes (*FMI*). E1, E1La and E1Lb also activate expression of *FT1a* and *FT4*, which encode flowering inhibitors. In long days, PhyA signaling also activates expression of *PRR3a* and *PRR3b*, which inhibit the expression of *CCA1*/*LHY*, which repress expression of E1. In short days, the evening complex (J/GmELF3, LUX, and likely GmELF4) inhibits the expression of the flowering repressors. Thus loss of J or LUX function delays flowering in short days, extending vegetative growth and increasing yield in the short days of the tropics. Arrows indicate positive regulation and lines with blunt ends indicate negative regulation. Please consult the main text for further details. Modified from [102].

*J* has been identified as the ortholog of Arabidopsis *EARLY FLOWERING3* (*ELF3*) [148], a component of the EC (Figure 3). *GmELF3* is suppressed by PHYA (encoded by *E3* and *E4*), and the GmELF3 protein physically binds to the promoter of *E1* near the LUX-binding motif to suppress *E1* transcription. This relieves the E1-dependent transcriptional repression of *FT2a* and *FT5a*, thereby promoting flowering. Multiple independent loss of function alleles of *GmELF3* (*J*) have been identified in low-latitude genotypes [148]. In LJ lines, with impaired *GmELF3* function, *E1* itself is released from repression and is able to repress *FT2a* and *FT5a*, resulting in later flowering. This permits increased vegetative growth and subsequently increased numbers of flowers, grain set, and yield [148]. This result is reminiscent of the observation that overexpression of an *Arabidopsis thaliana* B-box domain gene (*AtBBX32*) or its functional homologs, *GmBBX52* and *GmBBX53*, in transgenic soybean extended the duration of the pod and seed development period and thereby significantly increased soybean grain yield [149]. In Arabidopsis, *AtBBX32* is clock regulated and its overexpression in transgenic soybean alters transcript levels of the soybean clock genes *GmTOC1* and *LHY-CCA1-like2* (*GmLCL2*) around dawn (chiefly), although it has not been established that these changes in clock gene expression are either necessary or sufficient to confer the reproductive development changes leading to enhanced yield.

*ELF3* homologs are important controllers of flowering time in a number of other crops. For example, in chickpea, *Cicer arietinum*, spring flowering arises in landraces through mutations in *CaELF3a* [150]. *ELF3* homologs control short-day flowering in other legumes, including peas and lentils [151].

Other clock-related loci have been implicated in the control of flowering time. Elite cultivars of soybean exhibit a latitudinal cline in circadian period, with period lengthening at higher latitude [96]. Soybean has six cryptochrome-like (*CRY*) genes; one, *GmCRY1a*, is a strong promoter of flowering, and *GmCRY1a* exhibits a circadian rhythm in protein abundance that varies with latitude and correlates with photoperiodic flowering in long (but not in short) days [152].

In Arabidopsis, five PSEUDO-RESPONSE REGULATORS, important transcriptional repressors in the oscillator mechanism, are expressed sequentially from dawn until after dusk in the order, *PRR9*, *PRR7*, *PRR5*, *PRR3*, and *PRR1* (*TOC1*) [47]. In soybean, *GMPRR3a* and *GmPRR3b* have been shown to underlie growth period QTL, with extended growth period associated with increased yield [153]. In each case, the domesticated allele carries a loss of function (premature stop codon) mutation resulting in loss of the CCT domain [153] that is necessary for normal nuclear localization and DNA-binding [41,154]. Such loss of function alleles were associated with elevated expression of *GmFT2a* and *GmFT5a* and earlier flowering [153]. The frequency of this mutation of *GmPRR3b* increased from 5.6% in wild soybeans to 78.1% in landraces to 98.6% in improved cultivars, consistent with its selection during domestication and improvement [153]. Another study identified *GMPRR3a* and *GmPRR3b* as *Tof11* and *Tof12* and showed that they act as repressors of *GmLHY/CCA1* homologs (also called *GmLCL*) [155]. Soybean has four *GmLHY/CCA1* homologs that are clearly important for circadian clock function because a quadruple loss-of-function mutant has an extremely short period [155]. The *GmLHY/CCA1* homologs are repressors of *E1*. Thus, loss of *GmPRR3* function leads to upregulation of *GmLHY/CCA1*, downregulation of *E1* and, thereby, induction of *GmFT2a* and *GmFT5a* [156]. Conversely, the *Gmlhy/cca1* quadruple mutant fails to repress *E1* and flowers late [155]. *GmPRR3b* has also been associated with flowering time and maturity through a genome-wide association study [157]. One specific allele, *GmPRR3b^H6^* is predominant among modern soybean cultivars, consistent with selection during domestication. Its overexpression increases main stem node number and grain yield, and loss of function delays growth and flowering [157]. These effects on flowering may result from the repression of other clock genes, including *GmCCA1a*, via its regulation of *GmELF3a* (*J*).

A second *PRR* locus, a *PRR7* homolog, has been identified as a strong candidate for a flowering time QTL, qFT12-1, although there is no evidence establishing that different alleles of this locus alter clock function [158]. There are an additional nine *PRR* genes (a total of 12) in soybean, although there is to date no evidence linking these additional genes to domestication or improvement [153].

In Arabidopsis, considerable evidence links the circadian clock to both biotic and abiotic stress responses [12,159,160]. Clock function modulates responses to multiple stresses and, reciprocally, stresses modulate clock function, e.g., [161,162,163,164,165,166]. This reciprocal relationship extends to crops: the circadian clock modulates the drought response in poplar [167] and in *B. rapa* [168]. The soybean circadian clock has been shown to respond to multiple environmental stresses [169]. Two homologous pairs of *GmLHY/CCA1* genes were shown to be negative regulators of the drought response, and quadruple *Gmlhy/cca1* (more simply named quadruple *lhy)* loss of function mutants exhibit improved drought tolerance, likely through alteration of ABA signaling [170]. Thus, it seems possible that clock components such as *LHY* may provide multiple targets for improvement of crop stress responses, through either harnessing natural variation or via genome editing.

### 2.2. Tomato

Tomatoes (*Solanum lycopersicon*) offer an excellent example of clock function being subject to selection during domestication and improvement. The wild progenitor of the tomato (*Solanum pimpinellifolium*) originated in the Andes of Ecuador and Peru. Domestication occurred in two stages, first in South America and later in Central America [171]. The first step, in South America, was associated with the earliest domesticated Ecuadorian cherry tomatoes showing a delayed circadian phase relative to wild species [172]. The causal gene was shown to encode a phytochrome A–associated F-box protein homologous to Arabidopsis *EID1,* which is a negatively acting component of phytochrome A signaling [173]. The delayed phase phenotype is associated with a three bp deletion removing a conserved residue in the C terminus of the protein. Consistent with the cultivated *EID1* allele and altered circadian phase conferring an adaptive advantage**,** plants carrying the cultivated allele of *EID1* were shorter, flowered later and had higher chlorophyll content than those bearing the wild allele and the differences in chlorophyll content occurred specifically under long days [172]. The second step in domestication resulted in lengthened period resulting from a partial deletion of *LNK2* that, as described above, encodes a transcriptional coactivator in the clock [172,174]. In Arabidopsis, mutational disruption of *LNK2* function prevents transcriptional activation of *PRR5* by RVE8 and results in long circadian period [59]. LNK2, like EID1, contributes to light signaling to the circadian clock [59,175]. Both *EID1* and *LNK2* are located in chromosomal regions that exhibit very low genetic diversity, consistent with positive selection during tomato domestication or improvement. It seems reasonable to hypothesize that the slower and delayed phase clock represents an adaptation to long photoperiods encountered at higher latitudes, which may in turn enhance overall crop performance [172,174].

### 2.3. Sugar Beet

Sugar beet (*Beta vulgaris*) belongs to the Amaranthaceae family, whose lineage diverged from that of Arabidopsis shortly after the monocot–dicot split ~140 million years ago. Domestication of sugar beet occurred only within the past 200 years and domestication entailed a switch from an annual to a biennial habit with a requirement for vernalization, because bolting and flowering in the first year is associated with a drastic reduction in yield. Four loci, *B*, *B2, B3*, and *B5* have been identified as controlling bolting [176,177]. Of these, *B* has been shown to be a *PRR3/PRR7* homolog, *BOLTING TIME CONTROL 1* (*BvBTC1*) [178]. *BvBTC1* is necessary for flowering and mediates the response to both long days and vernalization through regulation of *BvFT* genes [179] and partial loss of function alleles of *BvBTC1* have been selected during domestication [178]. *B2* encodes a second transcription factor, B-BOX TYPE ZINC FINGER 19 (BvBBXC19), with both proteins necessary for CO-like activity and induction of the *FT* genes [180,181]. However, there is no experimental evidence establishing roles for these loci in circadian clock function in sugar beet.

### 2.4. Monocot Clocks

The examples discussed to this point have all been from eudicots, but similar pressures have been associated with range expansion among monocots. Cultivated grasses such as rice (*Oryza sativa*), maize (*Zea mays*), wheat (*T. aestivum*), and barley (*H. vulgare*) are enormously important crops. Although monocots have long been known to exhibit circadian regulation of gene expression, e.g., [182,183], the study of the molecular basis of monocot clocks has lagged behind that of eudicots. Phylogenetic analysis of the *PRR* and *CCA1*/*LHY* gene families shows that circadian clocks composed of multiple interlocked feedback loops evolved prior to the divergence of monocots and eudicots [184,185,186]. Orthologs of Arabidopsis clock genes have been identified in monocots such as rice [187,188], Lemna [189,190], other duckweeds [191], and barley [192,193,194,195].

Rice has a set of highly conserved clock-associated genes, including *OsCCA1*, *OsLUX*(*PCL*), five *OsPRR* genes including *OsTOC1*(*PRR1*), *OsZTLs*, and *OsGI* [187,188]. Similarly, barley has multiple orthologs to Arabidopsis clock genes, including *HvCCA1*, *HvRVE7* and *HvRVE8,* five *HvPRR* genes including *HvTOC1*(*PRR1*), *HvGI*, and *HvLUX*(*PCL*) [192,193,194,195].

In functional studies, loss of function of *OsGI* affected diurnal expression of 75% of all tested genes and conferred reduced seasonal adaptability in field-grown rice [196]. Rice orthologs of *TOC1* and *PRR7* partially complemented the corresponding Arabidopsis *toc1* and *prr7* mutants, which is consistent with the function of these proteins being conserved between monocots and Arabidopsis [197]. Recently it has been established that OsPRR73 is involved in a feedback loop of the rice clock and connects the circadian clock to the photoperiodic flowering pathway by binding to the *Ehd1* promoter as well as to the *OsLHY* promoter [198]. Loss of function of OsPRR73 results in early heading under LD but not SD, whereas overexpression results in late heading under both LD and SD [198]. Misexpression of OsPRR73 perturbs the expression of a number of clock genes.

In *Lemna gibba*, overexpression or RNAi-mediated downregulation of several genes (*LgLHYH1*, *LgLHYH2*, *LgGIH1*, and *LgELF3H1*) tested their roles in the circadian system. Overexpression of each gene and RNAi knock-downs of each of the genes except *LgLHYH2* disrupted the bioluminescence rhythms of clock reporter constructs [190].

There are several examples in which mutations of barley orthologs of Arabidopsis clock genes disrupt clock function. *HvPHYC* carrying a mutation in a conserved region of the GAF domain is a candidate underlying the *early maturity 5* (*eam5*) locus in barley [199]. *HvPHYC* interacts with *Ppd-H1* to accelerate flowering under noninductive short days. In addition, expression of a number of clock genes is perturbed in *eam5* mutants. Other barley early flowering mutants (*early maturity*; *eam*), *eam8* (allelic to *mat-a.8*) and *eam10*, carry mutations in *HvELF3* and *HvLUX1*, respectively [193,194,200]. As discussed below, these mutations facilitate adaption to short growing season, extending cultivation northward. Loss of function of *HvELF3* causes an up-regulation of *Ppd-H1* and the downstream *HvFT1* under noninductive SD conditions and results in severe perturbation of the expression of circadian clock genes [193]. Both the *Hvelf3* and *Hvlux1* loss of function mutants are arrhythmic and have lost circadian transcriptome oscillations under constant conditions [195]. *eam7*, another early flowering mutant whose identity has not yet been resolved, also exhibited severely perturbed clock function [195].

Collectively, these functional observations in Lemna, barley, and rice indicate that the structure of the circadian clock is likely to be conserved between monocots and eudicots.

### 2.5. Rice

Rice originated in the subtropics but is now cultivated over an expanded latitudinal range north to ~53°N and south to the tropics. Weakened photoperiod sensitivity is a critical factor for adaptation of rice to high-latitude regions. Combinations of weak alleles of *Ghd7*, *Hd1*, *PhyB*, as well as other important flowering determinants, act additively to reduce photoperiod sensitivity to enable rice cultivation in high latitude areas [201,202,203]. Similarly, allelic variants of flowering activation genes such as *Ehd4* and *RFT1* have also contributed to adaptation of rice to higher latitudes [204,205]. Given the importance of the circadian clock in the regulation of flowering time [70,76], discussed above, it seems quite possible to allelic variants of clock genes may contribute to improved performance and further range expansion.

### 2.6. Barley

Genetic variation in photoperiod response was also crucial for the successful expansion of barley cultivation from its origin in the Fertile Crescent to northern latitudes. A number of photoperiod insensitive *eam* loci, some of which correspond to clock genes, were mentioned above. However, the major determinant of the barley photoperiod response is the *HvPPR37* gene, *Ppd-H1*. Reduced photoperiod responsiveness of the *ppd-H1* mutant is highly advantageous in spring-sown varieties and results in late flowering, which can be explained by altered circadian expression of the photoperiod pathway genes *HvCO1* and *HvCO2*, delaying their diurnal expression peaks into the dark and so preventing accumulation of CO protein. This reduced CO accumulation results in reduced expression of its downstream target, *HvFT* (*Vrn-H3*) [206].

*PRR* genes feature prominently among regulators of flowering time (heading date) in the grasses. However, the evolution of the *PRR* family has differed in the grasses from that in the eudicots [185], and at least in some cases it seems that the genes regulating flowering time are distinct from those regulating the circadian clock. The *ppd-H1* mutation does not perturb circadian clock gene expression, suggesting that *Ppd-H1*(*PRR37*) does not contribute to circadian clock function [192]. This would be consistent with sub-functionalization among the barley *PRR* gene family in which *Ppd-H1*(*PRR37*) lost clock function, which was presumably retained by *HvPRR73*. Alternatively, functional redundancy between *Ppd-H1*(*PRR37*) and *HvPRR73* might prevent detection of a clock defect in the *ppd-H1* single mutant, although double mutant *ppd-H1 Hvprr73* might exhibit a more extreme phenotypic defect than the *Hvprr73* single mutant. For example, neither the *fkf1* not the *lkp2* single mutants of Arabidopsis exhibit clock defects, but both mutations enhance the long-period phenotype of the *ztl* mutant [207]. *Ppd-H1*(*PRR37*) is also a major determinant of leaf size in barley, likely via its induction of the MADS BOX genes *BM3* and *BM8* in the leaf [208].

## 3. Concluding Remarks

The importance of flowering time to crop performance has made it a critical target of efforts to expand latitudinal zones of cultivation. The centrality of the circadian clock to photoperiodic flowering has meant that allelic variation of circadian clock genes has contributed to range expansion in many crop species. However, the circadian clock contributes to plant performance in many ways beyond photoperiodic flowering. The circadian clock contributes to the regulation of circadian of growth and metabolism, as well as to abiotic and biotic stress responses [12,13,209]. As a consequence, there are many routes by which the circadian clock contributes to plant fitness and crop performance [11,210,211].

In Arabidopsis, a clock that resonates with the environmental daylength enhances photosynthesis and biomass accumulation [212]. An altered circadian clock in a new coffee clone has been correlated with higher photosynthesis efficiency and improved agronomic performance, although the mechanistic basis by which altered clock function does so remains uncertain [213]. Ni et al. (2009) showed that subtle changes in the temporal expression pattern of *CCA1* contributed to growth vigor in Arabidopsis hybrids and allopolyploids [214]. Epigenetic changes in circadian-related genes, including altered methylation of the promoter of the parental *CCA1* allele, have been shown to lead to biomass heterosis in Arabidopsis hybrids [215]. This has been extended from Arabidopsis to crops; early activation of CCA1-binding targets in maize hybrids promoted photosynthesis and biomass heterosis [216].

It seems likely that optimizing circadian function will continue to offer opportunities to enhance crop productivity, particularly in crops grown over broad latitudinal ranges. This need will be exacerbated by global warming which necessitates a poleward migration of zones of cultivation [217]. Efforts towards crop improvement to date have focused on standing allelic variation. However, improvements in genome editing now offer the potential to make precise targeted changes to the genome that are independent of standing variation [218]. Of course, such efforts towards genome editing need to be informed by greater understanding of the mechanistic means by which the clock enhances plant performance. The pervasive nature of clock influence mandates an informed approach to clock manipulation lest such efforts prove counterproductive. This offers a compelling rationale for the continued study of not only how the plant circadian clock keeps time, but also of how the plant uses that time information to regulate output pathways in a temporally dynamic fashion.
